# Organ-Specific Metabolome Reveals Potential Nutritional and Health Benefits of *Ampelopsis grossedentata*

**DOI:** 10.3390/metabo15090604

**Published:** 2025-09-10

**Authors:** Yanna Li, Ran Ye, Ju Yang, Siting Deng, Dongqing Rong, Yinling Luo, Hui Huang

**Affiliations:** 1 Key Laboratory of Research and Utilization of Ethnomedicinal Plant Resources of Hunan Province, College of Biological and Food Engineering, Huaihua University, Huaihua 418000, China; 15580152162@163.com (Y.L.); cutao487017126@163.com (R.Y.); 19974001279@163.com (J.Y.); 19118231181@163.com (S.D.); rongdongqing@gmail.com (D.R.); 2 Key Laboratory of Subtropical Medicinal Edible Resources, Development and Utilization in Yunnan Province, College of Biology and Chemistry, Puer University, Puer 665000, China; luoyinling@peu.edu.cn; 3 Yunnan Key Laboratory for Wild Plant Resources, Department of Economic Plants and Biotechnology, Kunming Institute of Botany, Chinese Academy of Sciences, Kunming 650201, China

**Keywords:** *Ampelopsis grossedentata*, vine tea, metabolic profiling, organ-specific, flavonoids, amino acid

## Abstract

**Background/Objectives:** *Ampelopsis grossedentata,* a novel food resource (vine tea), is a therapeutically valuable species wherein all parts exhibit bioactive potential. The therapeutic value and health benefits of *A. grossedentata* are rooted in its metabolomic profile, yet the metabolites in its various parts remain incompletely characterized. In this study, the tissue-specific biochemical properties and metabolic profiles of *A. grossedentata* were systematically characterized to identify the optimal tissue type for harvesting, with the goal of maximizing the production of bioactive compounds. **Methods:** The biochemical properties of various *A. grossedentata* tissues were assessed using a spectrophotometer, and their metabolic profiles were characterized through LC-MS/MS-based untargeted metabolomics. **Results:** The results demonstrate that, compared with the stems and roots, the tender leaves and floral tissues contained a higher TFC and TPC, correlating with superior antioxidant activity (DPPH, ABTS, and FRAP). A metabolic analysis revealed that the tender leaves accumulated multiple bioactive flavonoids beyond dihydromyricetin, including naringenin 7-O-beta-D-glucoside, (-)-epigallocatechin, tricetin, and cyanidin 3-O-sophoroside. The floral tissues displayed a comparable antioxidant capacity and dihydromyricetin level to the leaves, as well as unique compounds, such as leucodelphinidin, naringenin, epicatechin, dihydroquercetin, astilbin, and myricitrin. A metabolomic analysis further demonstrated a high accumulation of L-glutamine and L-glutamic acid in the floral tissues, potentially contributing to the characteristic umami flavor profile of vine tea made from *A. grossedentata* flowers. **Conclusions:** *A. grossedentata* flowers could be considered a promising raw material for developing novel functional foods and premium herbal tea products, as they possess superior antioxidant activity and abundant flavonoids and amino acids.

## 1. Introduction

In the commercial, academic, and governmental sectors, interest in functional food development has grown significantly in recent years. Herbal teas or tisanes, in particular, have gained global popularity as both a pleasurable beverage and a natural approach to health maintenance and disease prevention [1,2]. *Ampelopsis grossedentata* (Hand.-Mazz.), a perennial liana of the Vitaceae family, has been traditionally consumed as a functional herbal tea (commonly known as vine tea) in China. In 2013, the leaves of *A. grossedentata* were approved by the Chinese regulatory authority as a new food raw material. Moreover, all parts of *A. grossedentata* possess significant medicinal value and have been extensively utilized in traditional Chinese medicine, with historical records dating back to the Tang Dynasty [3,4]. Modern pharmacological studies have validated its diverse bioactive properties, including its antioxidant, anti-inflammatory, antidiabetic, antitumor, neuroprotective, and antibacterial properties, as well as its ability to alleviate hyperlipidemia [4,5,6]. Additionally, it has been reported that *A. grossedentata* leaf extract has natural antioxidants with the potential to extend the shelf life and guarantee the quality of foods such as edible oil, bread, and meat products [7].

*Ampelopsis grossedentata* is rich in specialized metabolites, including flavonoids, phenolic compounds, steroids, terpenoids, amino acids, water-soluble polysaccharides, and volatile compounds. These bioactive constituents, representing valuable natural sources of high-value phytochemicals, collectively determine both the therapeutic properties and unique flavor profile of vine tea [8]. Notably, flavonoids constitute the predominant class of secondary metabolites, accounting for up to 43% of total secondary compounds [9], with dehydromyricetin (DHM) being the most abundant flavonoid (reaching 30% by dry mass) [10]. Extensive studies have demonstrated that DHM exhibits various pharmacological activities, such as antioxidative, anti-inflammatory, antitumor, antibacterial, and antiviral liver protective activities, and that it can alleviate obesity and diabetes [4,11,12]. The abundant flavonoids and phenolic compounds, especially DHM, in vine tea also contribute to its characteristic frost-like leaf appearance and distinctive flavor profile, which features pronounced astringency, followed by a sweet aftertaste [4,11,12]. For instance, catechins and condensed tannins, as key flavonoid subclasses, serve as crucial determinants of both flavor characteristics and health-promoting properties, mirroring their well-established roles in *Camellia sinensis* [13]. Besides flavonoids, free amino acids have also been recognized as essential quality constituents, serving as primary contributors to the tea’s refreshing taste and mellow mouthfeel and as crucial precursors for volatile organic compounds (VOCs) [14]. Given that both therapeutic efficacy and tea quality are fundamentally determined by metabolite composition, comprehensive metabolomic profiling across the different development stages of the leaves and tissue types of *A. grossedentata* is imperative.

Recent years have witnessed a substantial increase in research investigating the chemical composition, functional properties, and health benefits of *A. grossedentata* leaves [4,6,11,12]. Significant attention has been directed toward elucidating the candidate genes (e.g., flavonoid biosynthesis genes, FLS, and DFR) involved in DHM biosynthesis [15,16,17,18]. Metabolomic studies have examined commercial vine tea products, including variations among three harvesting positions [19], characteristic flavor components [20], and dynamic metabolic changes during tea processing [21]. Despite these advances, the tissue-specific biochemical characteristics and metabolomic profiles of *A. grossedentata,* especially those of its flowers and roots, remain poorly characterized. Non-dominant tissues of medicinal plants have been reported to exhibit significant bioactivity yet remain substantially underutilized. For instance, although the roots of *Panax notoginseng* serve as the principal medicinal component, purified extracts of total saponins from its flowers can inhibit skin inflammation [22]. Both *Dendrobium officinale* and *D. devonianum* flowers contain flavonoids, amino acids, and fatty acids and have antioxidative activities, which should be explored for use in functional foods and pharmaceuticals [23]. Consequently, systematic metabolomic characterization provides critical insights to facilitate the comprehensive utilization of *A. grossedentata* resources, elucidate its underlying pharmacological mechanisms, and establish scientifically validated quality control standards for vine tea production.

Metabolomics has emerged as a powerful analytical platform for both biomarker discovery and phenotypic characterization, enabling the comprehensive identification of metabolites that modulate cellular and organismal functions [24]. The aims of the present study were to (1) characterize the organ-specific biochemical traits and metabolomic profiles of *A. grossedentata* and (2) establish optimal harvest parameters (including both plant parts and development stages) to maximize bioactive compound yield. Our findings provide critical insights for the utilization and quality control of vine tea resources, demonstrating how metabolomics can simultaneously facilitate bioactive compound screening and precision agriculture practices for this valuable medicinal plant.

## 2. Materials and Methods

### 2.1. Sample Collection

**Figure 1:**
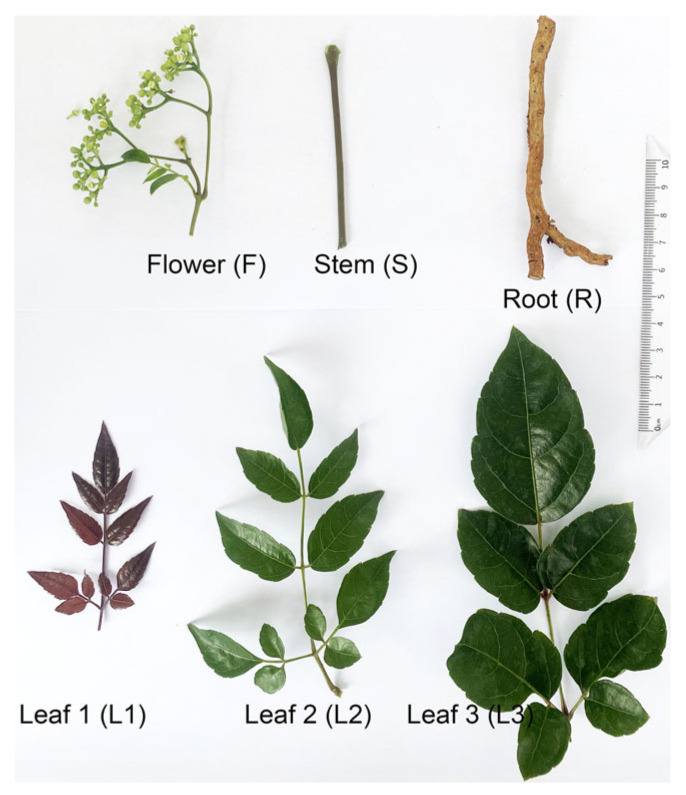
Phenotypic characteristics of *A. grossedentata* tissues used in this study.

*Ampelopsis grossedentata* materials (leaves, stems, roots, and flowers) were collected from plantations in Honghe, Yunnan Province, China (23.23° N, 102.25° E), in June 2023. The fresh tender tip leaves (L1), young green leaves (L2), mature green leaves (L3), stems (S), roots (R), and flowers (F) (Figure 1) were immediately frozen in liquid nitrogen and stored at −80 °C.

### 2.2. Measurement of Total Phenolic Content (TPC) and Total Flavonoid Content (TFC)

The TPC was determined using the Folin–Ciocalteu method described by Chen et al. [25]. For standard curve preparation, gallic acid solutions with different concentrations were aliquoted into cuvettes, followed by the sequential addition of 1 mL of Folin–Ciocalteu reagent and 2 mL of 12% Na_2_CO_3_ solution. The final volume was adjusted to 10 mL. After 1 h of incubation at 30 °C, the TPC was determined by measuring the absorbance at 765 nm using a UV spectrophotometer (Infinite M200 pro, Tecan, Männedorf, Switzerland). Distilled water served as a blank. The TFC was calculated using a modified version of the colorimetric method proposed by Xiao et al. [26]. Briefly, to generate a standard curve, rutin stock solution (0.02 ~ 1.5 mg/mL) was serially diluted to generate concentration gradients. Rutin solutions were mixed with 5% sodium nitrite and incubated at room temperature for 5 min, followed by the addition of 10% aluminum nitrate. Afterward, 4% sodium hydroxide was added, and the volume was adjusted to 10 mL using 60% ethanol. The solution mixture was then thoroughly mixed and incubated at room temperature for 12 min. The TFC was determined by measuring the absorbance at 510 nm using a UV–Vis spectrophotometer.

### 2.3. Measurement of Soluble Sugar, Amino Acid, and Soluble Protein Contents

The soluble sugar and amino acid contents were measured using a Plant Soluble Sugar Content Assay Kit and Amino Acid Content Assay Kit (Solarbio, Beijing, China). The soluble protein content was measured using specific detection kits (Nanjing Jiancheng Bioengineering Institute, Nanjing, China). The content is expressed as mg per g fresh weight (mg/g FW). All experiments were conducted in triplicate, and the results are presented as the mean ± standard error (SE).

### 2.4. DPPH Radical Scavenging Assay

The ability of plant extracts to scavenge 2,2-diphenyl−1-picrylhydrazyl (DPPH) free radicals was determined using specific detection kits (Nanjing Jiancheng Bioengineering Institute, Nanjing, China) according to the manufacturer’s instructions. A total of 40 μL of freshly prepared sample extract in methanol was added to 60 μL of DPPH solution. After 30 min of incubation in the dark at room temperature (RT ≈ 25 °C), the absorbance was measured at 517 nm using a UV–Vis spectrophotometer (Infinite M200 pro, Tecan, Männedorf, Switzerland). Methanol was used as a blank. A standard curve was prepared with different concentrations of Trolox (20 ~ 140 µM), and the results are expressed at μmol Trolox equivalents per g sample (μmol TE g^−1^).

### 2.5. ABTS Radical Cation Scavenging Activity

An ABTS assay was carried out using specific detection kits (Nanjing Jiancheng Bioengineering Institute, Nanjing, China) according to the manufacturer’s instructions. The [2,2’-azino-bis (3-ethylbenzthiazoline−6-sulfonic acid), ABTS] radical cation was made in 75 mM potassium phosphate-buffered saline solution (pH 7.4). Briefly, 5 µL of each sample and 195 µL of the ABTS radical cation solution were added to a tube and incubated for 6 min. Then, the absorbance was read at 734 nm. The control contained distilled water instead of sample extract. A standard curve was prepared using Trolox at different concentrations (0.15 ~ 1.5 mM), and the results are expressed as µmol of Trolox equivalents per g of sample (µmol TE g^−1^).

### 2.6. Ferric Reducing Antioxidant Power (FRAP)

A FRAP assay was performed using specific detection kits (Nanjing Jiancheng Bioengineering Institute, Nanjing, China) according to the manufacturer’s instructions. Briefly, the FRAP assay was performed by mixing 0.5 mL of ethanolic extract with 2.5 mL of freshly prepared FRAP reagent (10:1:1 ratio of 0.3 M acetate buffer [pH 3.6], 10 mM 2,4,6-tripyridyl-S-triazine [TPTZ], and 20 mM of ferric chloride hexahydrate [FeCl_3_.6H_2_O]). After pre-incubation at 37 °C for 10 min, the reaction mixture was incubated in the dark for 30 min. Absorbance was measured at 593 nm against ascorbic acid as a blank using a UV–Vis spectrophotometer.

### 2.7. Metabolomic Analysis

Metabolite extraction and a metabolomic analysis were carried out following our previous method [27]. The sample (0.1 g) was loaded into 600 µL of pre-chilled methanol, which contained 2-amino−3-(2-chloro-phenyl)-propionic acid (4 ppm), and it was ground for 90 s using a grinder. After ultrasound for 15 min, the mixture solution was centrifuged at 12,000 rpm and 4 °C for 10 min. The supernatant was filtered using a 0.22 µm membrane (nylon) and transferred into a detection bottle for UPLC-MS/MS detection.

Metabolomic detection was carried out on an ACQUITY UPLC System (Water, Milford, MA, USA), which was combined with an ACQUITY UPLC HSS T3 (150 × 2.1 mm, 1.8 µm) (Water, Milford, MA, USA) to separate the metabolic extracts. The raw mass spectrometry files generated by LC-MS were converted to mzXML file format using the MSConvert tool in the Proteowizard package (v3.0.8789) [28]. The XCMS package [29] was used for data processing, such as baseline filtering, peak area extraction and alignment, retention time correction, noise removal, and deconvolution. The mass spectrometry peak area data were normalized using Pareto scaling. At the same time, variables for QC samples with relative standard deviations (RSDs) > 30% were excluded, and log10 logarithmic processing was performed to obtain the final data matrix for subsequent analysis [30]. Additionally, the obtained MS/MS spectra data were matched against the authentic standards available in the metabolic public database HMDB (https://www.hmdb.ca/) (accessed on 21 May 2024), the Kyoto Encyclopedia of Genes and Genomes (KEGG) database [31], and the Metlin database (http://metlin.scripps.edu/) for the compound identification of metabolites. Multivariate analyses, including principal component analysis (PCA) and orthogonal partial least squares discriminant analysis (OPLS-DA), were performed using SIMCA software (Version16.0.2, Sartorius Stedim Data Analytics AB, Umea, Sweden), and a two-tailed Student’s *t*-test was conducted to obtain the variable influence in the projection (VIP) value and *p*-value of each metabolite. Metabolites with VIP value ≥ 1 and *p*-value < 0.05 were considered differentially abundant metabolites (DAMs).

### 2.8. Statistical Analysis

The experimental results are presented as the mean ± SE. Multi-factor and one-way ANOVAs (at 95% confidence) were performed, followed by Tukey’s multiple comparisons, using GraphPad Prism software (version 10.1.2) (GraphPad Software Inc., San Diego, CA, USA). Statistical significance was determined at *p* < 0.05.

## 3. Results

### 3.1. Biochemical Characteristics of Different A. grossedentata Tissues

**Figure 2:**
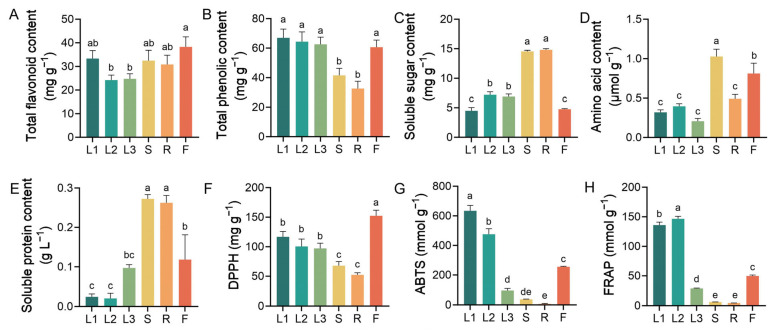
Total flavonoid (**A**), total phenolic (**B**), soluble sugar (**C**), amino acid (**D**), and soluble protein (**E**) contents and antioxidant activities, including DPPH (**F**), ABTS (**G**), and FRAP (**H**), in *A. grossedentata* tissues. Different letters indicate statistical differences according to Duncan’s multiple comparison test at *p* < 0.05. The fresh materials of the tender tip leaves (L1), young green leaves (L2), mature green leaves (L3), stems (S), roots (R), and flowers (F). All experiments were conducted in triplicate, and the results are presented as the mean ± SE.

This study revealed that the different tissues of *A. grossedentata* exhibited significantly different total flavonoid, total phenolic, soluble sugar, amino acid, and soluble protein contents and antioxidant activities (Figure 2). The highest TFC was found in the flowers (38.27 ± 3.80 mg g^−1^), followed by the tender leaves (33.34 ± 3.02 mg g^−1^) and stems (32.48 ± 3.91 mg g^−1^) (Figure 2A). The highest TPC was noted in the tender leaves (66.95 ± 5.32 mg g^−1^), followed by the young green leaves (64.38 ± 5.87 mg g^−1^) and mature green leaves (62.60 ± 4.34 mg g^−1^). Conversely, the lowest TPC and TFC was detected in the young green leaves (24.24 ± 1.89 mg g^−1^) and roots (32.65 ± 4.38 mg g^−1^), respectively (Figure 2B). The stems and roots contained significantly higher concentrations of soluble sugars and proteins than the leaf samples (L1, L2 and L3) (Figure 2C,E). There were abundant amino acids in the stems (1.03 ± 0.08 µmol g^−1^), followed by the flowers (0.81 ± 0.12 µmol g^−1^) and roots (0.49 ± 0.05 µmol g^−1^); the leaves showed the opposite trend, with a lower content of amino acids (Figure 2D). The tender and young green leaves and the flowers of *A. grossedentata* exhibited higher DPPH, ABTS, and FRAP antioxidant capacities than the stems and roots (Figure 2F–H). For instance, the ABTS and FRAP antioxidant activities of the tender leaves were 16 and 30 times higher those of the roots, respectively.

### 3.2. Metabolomic Profiling of Different Tissues of A. grossedentata

**Figure 3:**
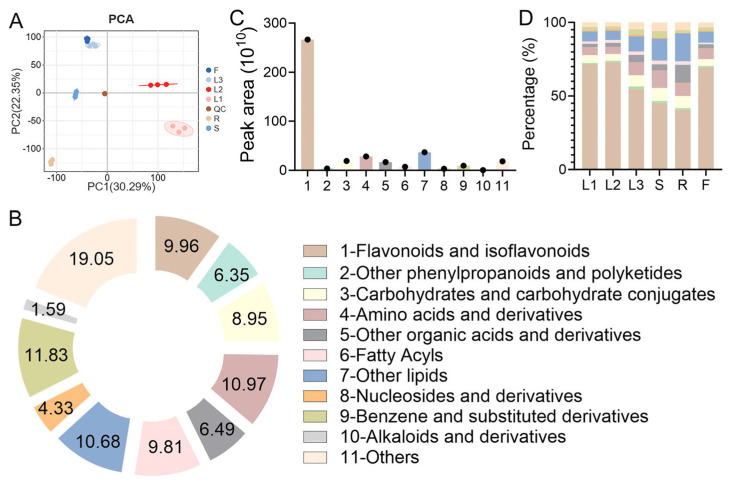
Metabolomic profiling of different tissues of *A. grossedentata.* (**A**) Principal component analysis (PCA). (**B**) Classification and composition of all identified metabolites. (**C**) Total peak areas of each metabolite class. (**D**) Proportion of total peak area of each compound class across different tissues. The color scheme for the compound categories in C and D is consistent with that in B. QC, quality, quality control. The fresh materials of the tender tip leaves (L1), young green leaves (L2), mature green leaves (L3), stems (S), roots (R), and flowers (F).

Non-targeted metabolomics using HPLC-MS/MS was carried out to comprehensively understand the metabolomic profiles of different tissues of *A. grossedentata.* A PCA analysis is illustrated in Figure 3A. PC1 and PC2 explained 30.26% and 22.35% of all metabolite variations, respectively. The cumulative variance explained by the first two PCs was 52.61%, indicating that they contained most of the information that could reflect all the data characteristics of the samples. The results indicate that there was significant divergence between the metabolomic profiling characteristics of the roots and other tissues. A total of 693 metabolites of 11 classes were identified, including 69 flavonoids and isoflavonoids; 44 other phenylpropanoids and polyketides; 62 carbohydrates and derivatives; 76 amino acids and analogues; 45 other organic acids and derivatives; 68 fatty acyls; 74 other lipids; 30 nucleosides, nucleotides, and analogues; 82 benzene and substituted derivatives; 11 alkaloids and derivatives; and 132 others (Figure 3B). Among these, benzene and substituted derivatives accounted for 11.83% of the total metabolites identified, followed by amino acids and derivatives (10.97%) and other lipids (10.68%). Furthermore, the summed peak areas of the 11 categories of compounds were computed individually (Figure 3C). The total peak area of the flavonoids and isoflavonoids was the highest among all compound classes, followed by that of other lipids and amino acids and derivatives. Figure 3D displays the percentage distributions of the 11 compound classes based on the summed peak areas in various tissues. The compositional profiles of the 11 metabolite classes showed remarkable similarity among the tender leaves, young green leaves, and flowers. The relative content percentage of flavonoids and isoflavonoids was significantly higher in the tender leaves, young green leaves, and flowers than in the mature green leaves, stems, and roots, which was the opposite to the relative content percentage of carbohydrates and carbohydrate conjugates, amino acids, peptides and analogues, and other lipids. Furthermore, the relative content percentage of flavonoids and isoflavonoids decreased with the maturation of the *A. grossedentata* leaves.

### 3.3. Identification and KEGG Enrichment Analysis of Differentially Abundant Metabolites

**Figure 4:**
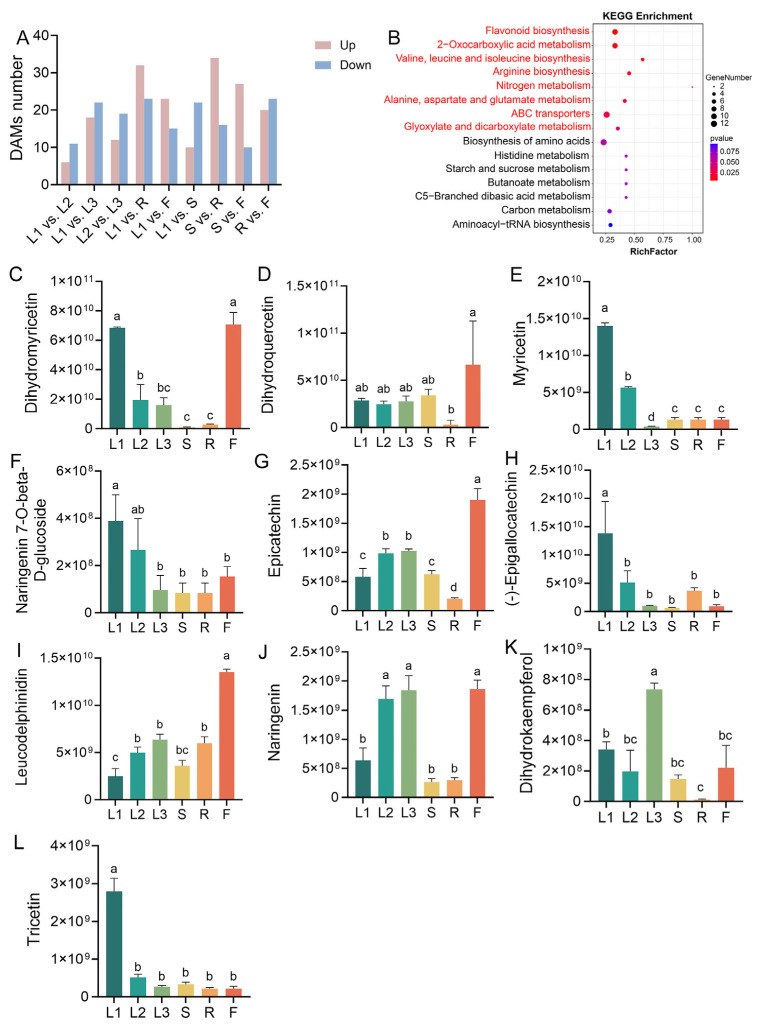
Identification and KEGG enrichment analysis of all DAMs. (**A**) Number of DAMs in each comparison group. (**B**) KEGG enrichment analysis of all DAMs. The metabolic pathways highlighted in red are significantly enriched pathways with *p* < 0.05. (**C**–**L**) The relative contents of metabolites enriched in the flavonoid biosynthesis pathway. The fresh materials of the tender tip leaves (L1), young green leaves (L2), mature green leaves (L3), stems (S), roots (R), and flowers (F). Different letters indicate statistical differences according to Duncan’s multiple comparison test at *p* < 0.05.

Using an OPLS-DA analysis, metabolites with VIP ≥ 1 and *p* < 0.05 were identified as DAMs in this study. Then, the number of DAMs in each comparison group was analyzed, and the results are visualized in Figure 4A. In total, 55 (L1 vs. R), 50 (S vs. R), 43 (R vs. F), 40 (L1 vs. L3), 38 (L1 vs. F), 37 (S vs. F), 32 (L1 vs. S), 31 (L2 vs. L3), and 17 (L1 vs. L2) DAMs were identified. The results show a significantly higher number of DAMs in the roots than in the other tissues, suggesting that the roots have the most distinct metabolic profile, which is in line with the results of the PCA of all metabolites. A KEGG enrichment analysis revealed a significant enrichment of DAMs in multiple metabolic pathways (*p* < 0.05), including flavonoid biosynthesis, 2-oxocarboxylic acid metabolism, branched-chain amino acid (valine, leucine, and isoleucine) biosynthesis, arginine biosynthesis, nitrogen metabolism, alanine/aspartate/glutamate metabolism, ABC transporters, and glyoxylate/dicarboxylate metabolism (Figure 4B). The relative contents of the metabolites enriched in the flavonoid biosynthesis pathway with the largest enrichment factor value are shown in Figure 4C–L. These metabolites displayed higher relative contents in the flowers and/or leaves than in the roots and stems. The tender leaves contained abundant DHM, myricetin, naringenin 7-O-beta-D-glucoside, (-)-epigallocatechin (EGC), and tricetin, while the flowers contained abundant DHM, dihydroquercetin, epicatechin (EC), leucodelphinidin, and naringenin. Dihydrokaempferol exhibited the highest relative content in the mature green leaves (Figure 4K). Most of the abovementioned flavonoids exhibited lower contents in the stems and roots than in the leaves.

**Figure 5:**
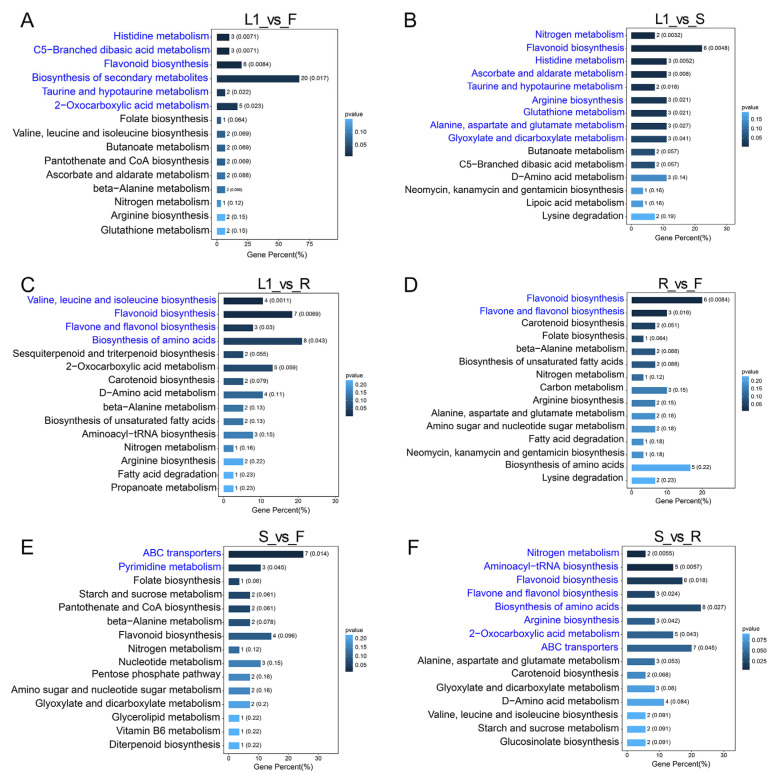
KEGG enrichment analysis of DAMs between different tissues. (**A**) L1 vs. F. (**B**) L1 vs. S. (**C**) L1 vs. R. (**D**) R vs. F. (**E**) S vs. F. (**F**) S vs. R. The metabolic pathways highlighted in blue are significantly enriched pathways with *p* < 0.05. The fresh materials of the tender tip leaves (L1), young green leaves (L2), mature green leaves (L3), stems (S), roots (R), and flowers (F).

An enrichment analysis facilitates the subsequent screening of pathways to identify those which demonstrate the highest association with diverse metabolites [32]. On this basis, different metabolites were linked to specific pathways in the KEGG database, and the major pathways of the different metabolites were obtained using an enrichment analysis. As shown in Figure 5A, the metabolic pathways enriched in DAMs between the tender leaves and flowers were involved in histidine metabolism, C5-branched dibasic acid metabolism, flavonoid biosynthesis, secondary metabolite biosynthesis, taurine and hypotaurine metabolism, and 2-oxocarboxylic acid metabolism. In the L1 vs. S comparison, the DAMs were mainly enriched in the pathways of nitrogen metabolism; flavonoid biosynthesis; histidine metabolism; ascorbate and aldarate metabolism; taurine and hypotaurine metabolism; arginine biosynthesis; glutathione metabolism; alanine, aspartate, and glutamate metabolism; and glyoxylate and dicarboxylate metabolism (Figure 5B). Conversely, the DAMs between the tender leaves and roots were enriched in branched-chain amino acid (valine, leucine, and isoleucine) biosynthesis, flavonoid biosynthesis, flavone and flavonol biosynthesis, and amino acid biosynthesis pathways (Figure 5C). Between the roots and flowers, the DAMs were enriched in flavonoid biosynthesis and flavone and flavonol biosynthesis pathways (Figure 5D). Many DAMs involved in ABC transporters and pyrimidine metabolism were found in the comparison of the stems and flowers (Figure 5E). In the comparison of the stems and roots, the primary enrichment pathways were as follows: nitrogen metabolism, aminoacyl-tRNA biosynthesis, flavonoid biosynthesis, flavone and flavonol biosynthesis, amino acid biosynthesis, arginine biosynthesis, 2-oxocarboxylic acid metabolism, and ABC transporters (Figure 5F). Notably, the flavonoid biosynthesis pathway was enriched in most of the comparisons, except for S vs. F, suggesting its significant divergence between different tissues of *A. grossedentata.* Moreover, the amino acid biosynthesis pathway was enriched in the DAMs between the leaves and other tissues, indicating its important role in determining the nutrition and quality of vine tea.

### 3.4. Clustering Analysis of DAMs

**Figure 6:**
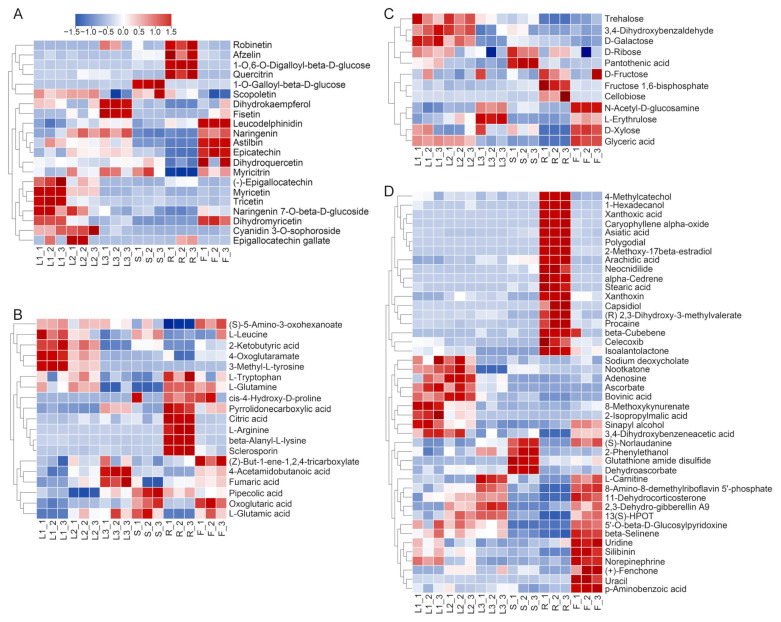
Clustering heatmaps of DAMs. (**A**) Phenylpropanoids and polyketides. (**B**) Organic acids and derivatives. (**C**) Carbohydrates and carbohydrate conjugates. (**D**) Other metabolites. The fresh materials of the tender tip leaves (L1), young green leaves (L2), mature green leaves (L3), stems (S), roots (R), and flowers (F).

High-frequency clustering heatmaps are important tools used to demonstrate the similarity between metabolites and samples. As shown in Figure 6, the samples were grouped and classified via hierarchical clustering of the metabolite levels in different tissues of *A. grossedentata*. Besides the flavonoids in Figure 4C–L, the tender leaves and young green leaves contained abundant cyanidin 3-O-sophoroside and epigallocatechin gallate (EGCG), while the flowers contained abundant astilbin and myricitrin. Dihydrokaempferol and fistein were abundant in the mature green leaves. Robinetin, 1-O,6-O-digalloyl-beta-D-glucose, afzelin, and quercitrin exhibited higher relative contents in the roots, while 1-O-galloyl-beta-D-glucose was rich in the stems (Figure 6A).

Among the DAMs belonging to the category of organic acids and derivatives, L-leucine, 2-ketobutyric acid, 4-oxoglutaramate, and 3-methyl-L-tyrosine exhibited higher relative contents in the tender leaves and young green leaves (Figure 6B), while L-tryptophan and L-glutamine exhibited the highest relative contents in the roots, followed by the young green leaves, tender leaves, and flowers. Citric acid, L-arginine, beta-alanyl-L-lysine, and sclerosporin exhibited higher relative contents in the roots. 4-acetamidobutanoic acid and fumaric acid were abundant in the mature green leaves and flowers. Pipecolic acid, oxoglutaric acid, and L-glutamic acid were rich in the stems, followed by in the flowers and mature green leaves.

As shown in Figure 6C, trehalose, D-galactose, and 3,4-dihyroxybenzaldehyde were abundant in the tender and young green leaves. D-ribose and pantothenic acid displayed higher relative contents in the stems, followed by in the tender leaves, while D-fructose, fructose 1,6-bisphosphate and cellobiose were enriched in the roots. The flowers contained higher contents of L-erythrulose and D-xylose. Glyceric acid was enriched in the flowers and tender and young green leaves. Additionally, the majority of terpenoids showed preferential accumulation in the roots (xanthoxin, asiatic acid, xanthoxic acid, isoalantolactone, capsidiol, beta-cubebene, alpha-cedrene, and caryophyllene alpha-oxide) and flowers (2,3-dehydro-gibberellin A9, beta-selinene, and fenchone) (Figure 6D).

### 3.5. Correlation Analysis

**Figure 7:**
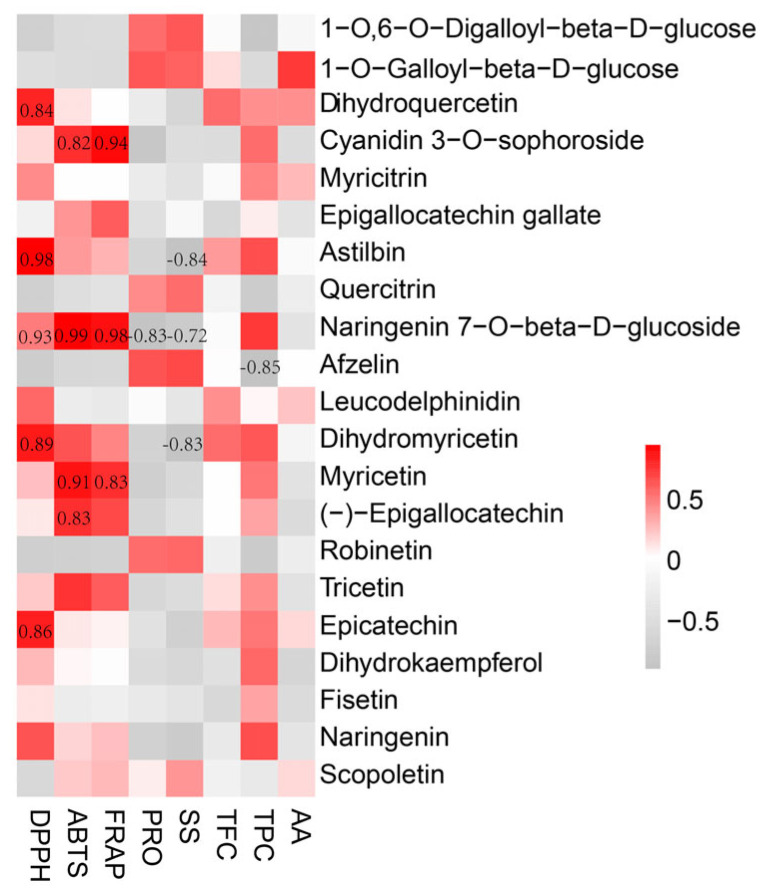
Correlation analysis of differentially abundant metabolites related to phenylpropanoids and polyketides with the TPC, TFC, amino acid content, soluble sugar content, soluble protein content, and antioxidant capacities (DPPH, ABTS, and FRAP).

Flavonoids constitute the predominant class of secondary metabolites in *A. grossedentata*, exhibiting an excellent antioxidant capacity and various pharmacological activities. To explore the internal relationships, we conducted Pearson’s correlation analysis between the DAMs belonging to the category of phenylpropanoids and polyketides and the TPC, TFC, amino acid content, soluble sugar content, soluble protein content, and antioxidant capacities (DPPH, ABTS, and FRAP). To identify metabolites significantly contributing to biological activities, we selected those demonstrating a relevance coefficient of *p* < 0.05. As evident in Figure 7, twelve bioactive compounds, namely, dihydroquercetin, cyanidin 3-O-sophoroside, EGCG, astilbin, naringenin 7-O-beta-D-glucoside, DHM, myricetin, EGC, tricetin, EC, dihydrokaempferol, and narigenin, exhibited positive correlations with antioxidant activities (assessed using DPPH, ABTS, and FRAP assays) and the TPC. Among these compounds, dihydroquercetin, cyanidin 3-O-sophoroside, astilbin, naringenin 7-O-beta-D-glucoside, DHM, myricetin, EGC, and EC exhibited significantly higher positive correlation coefficients (CC > 0.8), indicating their crucial contribution to the enhanced antioxidant capacities and bioactive properties of *A. grossedentata.* Notably, the abovementioned phenylpropanoid and polyketide compounds showed negative correlations with the soluble protein and soluble sugar contents. Conversely, 1-O,6-O-digalloyl-beta-D-glucose, 1-O-galloyl-beta-D-glucose, quercitrin, afzelin, robinetin, and scopoletin showed positive correlations with the soluble protein and soluble sugar contents. Additionally, 1-O-galloyl-beta-D-glucose, dihydroquercetin, myricitrin, leucodelphinidin, EC, and scopoletin showed positive correlations with the amino acid content.

## 4. Discussion

As a medicinal plant with all of its parts demonstrating therapeutic value, the health benefits of *A. grossedentata* are derived from the bioactive compounds distributed throughout its tissues. To systematically elucidate its potential nutritive value and health-promoting effects, we conducted the first comprehensive investigation of its tissue-specific biochemical traits and metabolic profiles.

Tender leaves with abundant flavonoids have generally been used to produce top-grade vine tea, which exhibits high nutritional value and a strong sweet aftertaste and astringency [5]. In this study, the highest DHM and myricetin contents were found in the tender leaves, which is in line with the results obtained by Wang et al. [19]. Both of these flavonoids had a strong correlation with the medicinal properties and flavor characteristics of vine tea. Myricetin is a critical nutritive dietary component that provides immunological protection and is beneficial for maintaining good health [33]. Moreover, several other flavonoids, including EGC, naringenin 7-O-beta-D-glucoside, tricetin, and cyanidin 3-O-sophoroside, were also rich in the tender leaves. Naringenin−7-O-glucoside has been confirmed to protect or alleviate doxorubicin cardiotoxicity by activating endogenous defense systems and anti-apoptosis pathways [34]. Tricetin and cyanidin 3-O-sophoroside also exhibit diverse bioactivities, such as antioxidant and anti-inflammatory effects, and they have potential therapeutic applications [35,36].

Notably, the flowers, stems, and roots exhibited significantly higher TFCs than both the young and mature green leaves, with floral TFC levels approximating those observed in the tender leaves. Furthermore, *A. grossedentata* flowers also contained a higher TFC than the leaf tissues. This parallel accumulation of phenolic compounds was strongly associated with superior antioxidant capacity (DPPH/ABTS/FRAP) in both the floral and foliar tissues. Moreover, DHM was abundant in the flower tissues compared to in the foliar tissues. Jia et al. [37] reported that the reducing capability of tender tip leaf extracts was higher than that of young green leaves and mature green leaves, which is consistent with our findings. Indeed, the flowers of many medicinal plants contain various bioactive compounds and exhibit higher antioxidant activities and more health benefits, such as those of *P. notoginseng* [22], *D. officinale* and *D. devonianum* [23], and *Salvia miltiorrhiza* [38]. Many other flavonoids, including leucodelphinidin, naringenin, astilbin, EC, dihydroquercetin, and myricitrin, were also identified in the *A. grossedentata* flowers. Dihydroquercetin is a unique bioactive flavonoid and considered a new food with good market prospects in many countries [39]. It can alleviate acute alcoholic liver injury and improve skin repair and skin inflammation [40]. Astilbin, a dihydroflavonol rhamnoside widely distributed in various plant species, demonstrates broad-spectrum pharmacological potential, including potent antioxidant, antidepressant, antimicrobial, and insecticidal activities. Notably, astilbin has shown neuroprotective properties by enhancing central nervous system cell function through multiple mechanisms [41]. Moreover, the significantly lower levels of EGCG and EGC in the *A. grossedentata* flowers than in the tender leaves make it more suitable for producing high-quality vine tea with a reduced bitter taste. Catechins, especially EGCG, are positively correlated with the bitterness of green tea [42]. The unique phytochemical profile of *A. grossedentata* flowers, characterized by an elevated TPC and TFC, coupled with a potent antioxidant capacity, suggests its significant potential as a premium botanical resource for functional food development and high-value herbal tea formulations.

Compared with the stems and roots, the young and mature green leaves of *Ampelopsis grossedentata* exhibited a lower TFC; a higher TPC; and higher DPPH, ABTS, and FRAP antioxidant capacities. The results indicate that, along with flavonoids, other phenolic compounds (e.g., phenolic acids) contribute to the antioxidant capacity of the young and mature green leaves. For instance, trans-ferulic acid and 4-hydroxycinnamic acid, both belonging to the category of phenolic acids, were enriched in the young and mature green leaves. Trans-ferulic acid is a derivative of 4-hydroxycinnamic acid, which is found in many food products, fruits, and beverages. It has scientifically proven antioxidant, anti-inflammatory, and antibacterial properties [43]. Hydroxycinnamic acids and their derivatives act as powerful antioxidants and protect biologically important molecules from oxidation [44].

Flavonoid biosynthesis in different tissues of *A. grossedentata* is governed by a complex network of structural and regulatory genes. Many flavonoid biosynthesis genes were highly expressed in the tender leaves (*PAL, C4H, 4CL, CHS, CHI, F3H, F3’H,* and *F3’5’H*) and roots (*PAL, C4H, F3H, F3’H,* and *CPR*) [17], which may contribute to the abundant flavonoids in the leaves and roots. A metabolomic analysis revealed that the DAMs in the *A. grossedentata* tissues were predominantly enriched in the flavonoid biosynthesis pathway, with floral tissues exhibiting the highest accumulation. This pattern likely results from the elevated expression of both structural and regulatory genes associated with flavonoid biosynthesis. Future research should integrate gene expression analysis with functional validation to accurately elucidate the underlying mechanisms.

The majority of free amino acids in tea exhibit distinct odor characteristics and threshold values [45]. Elucidating the distribution patterns of free amino acids across different tissues of *A. grossedentata* would establish a theoretical framework for targeted quality improvement and flavor modulation in vine tea production. The taste sensory results of an amino acid-simulated solution and green tea infusion demonstrated that, in green tea, L-glutamine, L-glutamic acid, and L-aspartic acid might contribute to the umami taste; L-alanine and D-phenylalanine might promote the sweet characteristic; and L-phenylalanine and L-tryptophan might be involved in the formation of bitterness [21]. In *A. grossedentata*, both the stems and flowers contained abundant total amino acids, though their accumulation profiles exhibited significant tissue-specific variations. The abundant L-glutamine and L-glutamic acid in *A. grossedentata* flowers may provide vine tea made from these parts with an umami taste. Conversely, the high accumulation of aromatic amino acids (L-phenylalanine, L-tryptophan, and L-tyrosine) in tender leaves, young green leaves, and floral tissues may contribute to both the characteristic bitter taste and the formation of complex volatile profiles in these tissues. Moreover, gamma-aminobutyric acid (GABA), a non-proteinaceous amino acid, exhibited higher levels in the tender leaves, mature green leaves, and flowers. GABA is widely used as a functional ingredient in functional foods or nutraceuticals due to its multiple physiological functions, such as its antihypertensive, antianxiety, and immunity enhancement effects and blood pressure regulation activity [46,47]. Additionally, higher soluble sugar and protein contents were observed in the stems and roots than in the leaves. Leaves are typically rich in photosynthetic proteins such as rubisco. The high soluble protein content in the stems and roots observed here might be attributed to the abundance of specific storage proteins or defense-related proteins, especially in nitrogen-fixing plants, which warrants further proteomic investigation. Moreover, it is well established that environmental stressors can significantly alter metabolomic profiles, potentially enhancing the production of certain defensive compounds (e.g., phenolics, flavonoids, and soluble sugar) while reducing that of others [48,49]. Our study reveals the unique metabolic profiles of different tissues of *A. grossedentata* and offers insights into the link between metabolites, medicinal value, and tea flavor.

## 5. Conclusions

By comparing the biochemical properties and metabolomic profiles of different tissues of *A. grossedentata*, our results reveal that the tender leaves and flowers contain a higher TPC and TFC and exhibit a significantly higher antioxidant capacity than the mature green leaves, stems, and roots. This remarkable antioxidant potential of *A. grossedentata* flowers is attributed to their higher contents of phenolics and flavonoids, such as naringenin 7-O-beta-D-glucoside (CC > 0.9) and myricetin (CC > 0.8), which play a crucial role in neutralizing free radicals and reducing oxidants. The results suggest that *A. grossedentata* flowers could be considered a promising raw material for the development of novel functional foods and premium herbal tea products.

## Data Availability

Data are contained within the article and Supplementary Materials.

## Supplementary Materials

The following supporting information can be downloaded at https://www.mdpi.com/article/10.3390/metabo15090604/s1. Table S1: Metabolites identified in various parts of *Ampelopsis grossedentata*. Table S2: Differentially accumulated metabolites among various parts of *Ampelopsis grossedentata*.

## Abbreviations

F3H, flavanone 3-hydroxylase; F3’H, flavonoid 3’-hydroxylase; F3’5’H, flavonoid 3’,5’-hydroxylase; CPR, cytochrome P450 reductase; PAL, phenylalanine deaminase; C4H, cinnamate 4-hydroxylase; 4CL, 4-coumarate-CoA ligase; CHS, chalcone synthase; CHI, chalcone isomerase; DHM, dehydromyricetin; VOC, volatile organic compound; FLS, flavonol synthase; DFR, dihydroflavonol 4-reductase; TPC, total phenolic content; TFC, total flavonoid content; DPPH, 2,2-diphenyl−1-picrylhydrazyl; ABTS, 2,2’-azino-bis (3-ethylbenzthiazoline−6-sulfonic acid); TPTZ, 2,4,6-tripyridyl-S-triazine; FRAP, ferric reducing antioxidant power; RSD, relative standard deviation; KEGG, Kyoto Encyclopedia of Genes and Genomes; PCA, principal component analysis; OPLS-DA, orthogonal partial least squares discriminant analysis; DAM, differentially abundant metabolite; VIP value, variable influence in the projection value; EGC, (-)-epigallocatechin; EC, epicatechin; EGCG, epigallocatechin gallate; GABA, gamma-aminobutyric acid.
